# Nab-Paclitaxel in the Treatment of Gastrointestinal Cancers—Improvements in Clinical Efficacy and Safety

**DOI:** 10.3390/biomedicines11072000

**Published:** 2023-07-15

**Authors:** Md Sazzad Hassan, Niranjan Awasthi, Saisantosh Ponna, Urs von Holzen

**Affiliations:** 1 Department of Surgery, Indiana University School of Medicine, South Bend, IN 46617, USA; 2 Harper Cancer Research Institute, South Bend, IN 46617, USA; 3 Department of Chemistry and Biochemistry, University of Notre Dame, South Bend, IN 46556, USA; 4 Goshen Center for Cancer Care, Goshen, IN 46526, USA; 5 Department of Surgery, University of Basel School of Medicine, 4001 Basel, Switzerland

**Keywords:** nab-paclitaxel, paclitaxel, efficacy, safety, toxicity, overall response rate (ORR), overall survival (OS), progression-free survival (PFS)

## Abstract

Taxanes (paclitaxel and docetaxel) are one of the most useful classes of anticancer drugs. Taxanes are highly hydrophobic; therefore, these drugs must be dissolved in organic solvents (polysorbate or Cremophor EL), which contribute to their toxicities. To reduce this toxicity and to enhance their efficacy, novel formulations have been developed. Nanoparticle albumin-bound paclitaxel (nab-paclitaxel) is an albumin-stabilized, Cremophor-free, and water-soluble nanoparticle formulation of paclitaxel. Nab-paclitaxel has better solubility and less infusion-associated toxicity compared to solvent-based paclitaxel. Additionally, nab-paclitaxel can be given at higher doses and concentrations compared with solvent-based paclitaxel. Based on its superior clinical efficacy and safety profile, nab-paclitaxel received FDA approval for metastatic breast cancer (2008) and NSCLC (2011). Among gastrointestinal cancers, it is now approved in the USA for treating patients with metastatic adenocarcinoma of the pancreas as first-line therapy in combination with gemcitabine. Furthermore, several clinical trials have suggested the potential efficacy of nab-paclitaxel as a single agent or in combination with other agents for the treatment of metastatic esophageal, gastric, bowel, and biliary tract cancers. Nab-paclitaxel has been demonstrated to have greater overall response rates (ORR) with enhanced progression-free survival (PFS), overall survival (OS) and a superior safety profile with fewer adverse effects in patients with gastrointestinal tract cancers. This review summarizes the advantages associated with nab-paclitaxel-based regimens in terms of improving clinical efficacy and the safety profile in upper gastrointestinal cancer.

## 1. Introduction

The taxanes paclitaxel and docetaxel are among the most important antineoplastic drugs that function as microtubule-stabilizing agents, leading to the inhibition of cell mitosis. Paclitaxel and docetaxel were first approved by the Food and Drug Administration (FDA) for clinical use in the United States (US) in 1994 and 2004, respectively [1,2]. These taxanes are extensively used to treat various cancers, including gastrointestinal cancers such as esophageal, gastric, and pancreatic cancers. Although these taxanes are clinically very effective in treating a variety of cancers, they have very serious adverse effects that often hinder their regular use. The solvents used to dissolve taxanes have a major contribution towards toxicities associated with taxanes [3,4]. Since taxanes are hydrophobic, Cremophor EL (CrEL) is used to dissolve paclitaxel, and polysorbate-80 is used to dissolve docetaxel [4]. These solvents contribute to considerable adverse effects such as hypersensitivity reactions, including anaphylactic reactions and neuropathies [3,4]. Additionally, these solvents impair tumor penetration, limiting the clinical effectiveness of paclitaxel and docetaxel [3,4]. Furthermore, the uptake of taxanes by cells is impaired by P-glycoprotein, which actively exports paclitaxel out of the cells, leading to paclitaxel resistance [5,6].

**Figure 1:**
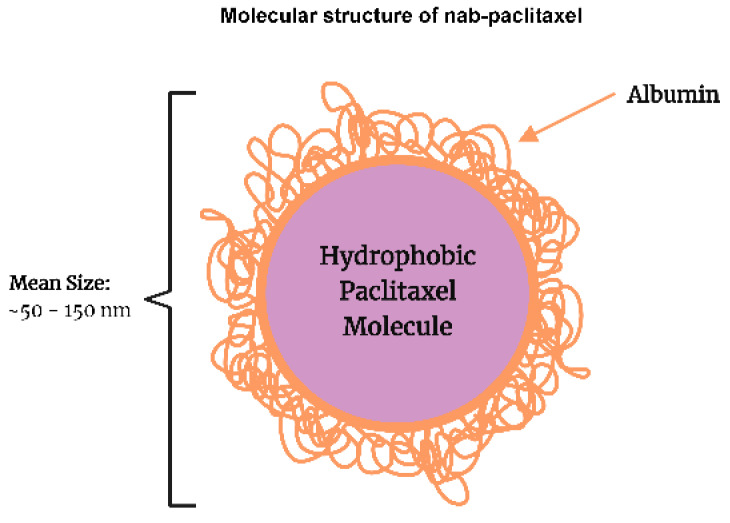
Molecular Structure of nab-paclitaxel.

**Figure 2:**
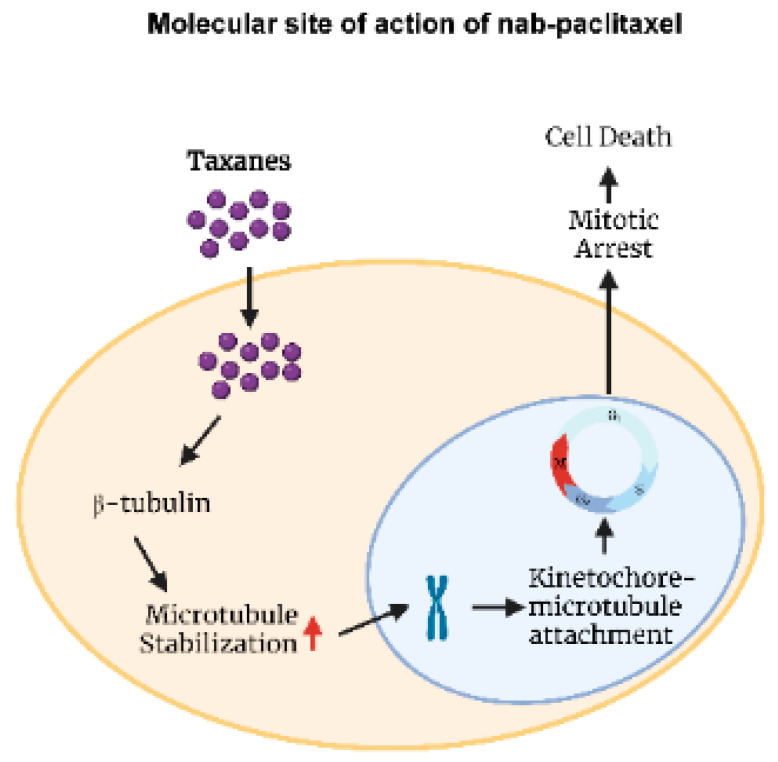
Molecular site of action of nab-paclitaxel.

**Figure 3:**
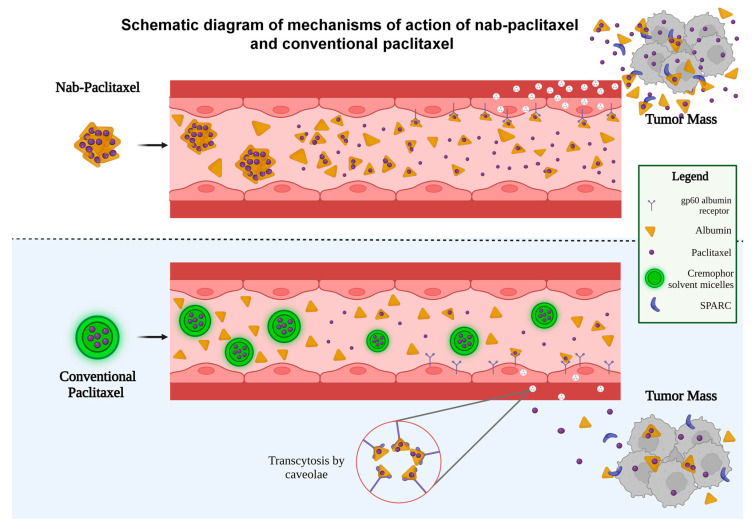
Schematic diagram of mechanisms of action of nab-paclitaxel and conventional paclitaxel.

Due to the significant toxicities associated with the clinical use of solvent-based taxanes, numerous efforts have been made to overcome this issue. The development of a nanoparticle albumin-bound formulation of paclitaxel (nab-paclitaxel) is a result of these efforts. Nab-paclitaxel is a colloidal suspension of Cremophor solvent-free albumin-bound nanoparticle (mean diameter ~130 nm) of paclitaxel, which is homogenized with human serum albumin in an aqueous solution [7]. This water soluble nab-paclitaxel has appeared as the favored treatment option due to its superior clinical efficacy and lower incidence of toxicity compared to solvent-based paclitaxel [8]. The tolerable dose of nab-paclitaxel is much higher than paclitaxel with fewer side effects [7]. In a mouse xenograft model, it has been shown that the maximum tolerable dose (MTD) of nab-paclitaxel was much higher (30 mg/kg/day) than that of cremophor solvent-based paclitaxel (13 mg/kg/day) [9]. Similarly, in humans, the MTD of nab-paclitaxel was higher (300 mg/m^2^, every three weeks) compared to that of solvent-based paclitaxel (250 mg/m^2^, every three weeks) with significantly fewer adverse effects [10,11]. Nab-paclitaxel (Abraxane^®^) was approved by the FDA in the USA in January 2005 for use in patients with metastatic breast cancer and in October 2012 for use in patients with locally advanced or metastatic non-small cell lung cancer (NSCLC) [12,13]. Nab-paclitaxel, in combination with gemcitabine, is also approved in the USA for treating advanced pancreatic cancer [14]. The development of nab-paclitaxel became possible with the advent of the medical applications of drug nanotechnology. Drug nanotechnology involves the design and development of novel drugs that carry nanoparticles (Figure 1) [15]. The molecular structure of nab-paclitaxel, as depicted graphically in Figure 1, consists of a hydrophobic center containing paclitaxel due to its lack of solubility in water. The external hydrophilic shell is made of human serum albumin, which has negatively charged amino acids. The size of the molecule ranges from 50 to 150 nm, with a mean diameter of around 130 nm. The negatively charged amino acids of albumin repel each other, keeping the molecules in a homogenous aqueous suspension. Nab-paclitaxel essentially functions similar to solvent-based paclitaxel and other taxanes by binding to a pocket in the β-tubulin, thereby disrupting microtubule organization leading to the suppression of cell mitosis, as shown graphically in Figure 2. With this new albumin-bound formulation, nab-paclitaxel can cross endothelial cells by binding with an albumin receptor known as gp60, which is expressed on the surface of the endothelium, as shown graphically in Figure 3 [16]. Thus, unlike solvent-based paclitaxel, nab-paclitaxel exploits albumin to carry paclitaxel directly into cancer cells through a receptor-utilized carrier system, enhancing paclitaxel distribution at the tumor site [16]. As graphically shown in Figure 3, the binding of albumin with the gp60 receptor stimulates caveolin-1, resulting in the formation of vesicles known as caveolin, which facilitate the transport of the albumin-drug complex into the tumor interstitial space through transcytosis [16]. Consequently, there is an enhanced intratumoral nab-paclitaxel accumulation due to its enhanced binding and transport [9]. Once nab-paclitaxel is in the extravascular space, it can accumulate inside the tumor through the interaction between SPARC (secreted protein acidic and rich in cysteine) and albumin. SPARC is present in the tumor microenvironment of many cancers including gastrointestinal cancers [17,18] (Figure 3). It has been demonstrated that SPARC promotes intratumoral aggregation of albumin-bound nanoparticles [19], and nab-paclitaxel utilizes this characteristic of cancer biology, as it is preferentially retained by tumoral SPARC.

This article provides a review of nab-paclitaxel’s role in improving clinical efficacy and safety during the treatment of gastrointestinal (GI) cancers.

## 2. Pharmacokinetics of Nab-Paclitaxel

Nab-paclitaxel has been shown to improve the pharmacokinetics and biodistribution of paclitaxel compared to solvent-based paclitaxel (sb-paclitaxel) in solid cancers [20,21]. In these studies, the maximum plasma concentration (*C_max_*) of paclitaxel was 3.8 to 6.5 folds higher after infusion of nab-paclitaxel compared to sb-paclitaxel [20,21]. In phase I clinical trials, the plasma pharmacokinetics (PK) of paclitaxel or gemcitabine were compared between nab-paclitaxel (125 mg/m^2^) monotherapy and nab-paclitaxel (125 mg/m^2^) plus gemcitabine (1000 mg/m^2^) combination therapy [22,23]. The results showed no significant differences in peak plasma concentration (*C_max_*), clearance (CL), volume distribution (Vz) or half-life (*t*_1/2_) of either paclitaxel or gemcitabine between the treatments [22,23]. In another phase I pharmacokinetics study, where nab-paclitaxel was used in combination with oral fluoropyridine S-1 [24], the PK parameters of the drugs were very similar when nab-paclitaxel was administered either alone or co-administered with S-1 [24]. These results indicate that nab-paclitaxel does not have any significant drug interactions when used in combination with other anticancer therapies. In another phase I PK study, nab-paclitaxel was administered in patients with solid tumors, including GI cancers, through hepatic atrial infusion (HAI) to compare the hepatic extraction of nab-paclitaxel between hepatic arterial and intravenous infusions [24]. Hepatic extraction of nab-paclitaxel represents the amount of nab-paclitaxel directly released to the liver, bypassing systemic vulnerability. In this study, approximately 42% higher hepatic extraction of nab-paclitaxel was observed following HAI compared to intravenous infusion, indicating that HAI can achieve better antitumor activity in patients with liver metastasis [24].

## 3. Nab-Paclitaxel in Pancreatic Cancer

Pancreatic ductal adenocarcinoma (PDAC) has the highest mortality rate among all major cancers. PDAC has a poor prognosis with a 5-year survival rate of <10% for all stages combined [25]. Gemcitabine (Gem) has remained the standard treatment for advanced PDAC patients since 1997; however, it only demonstrated a modest clinical response with a median survival of approximately 6 months [26,27]. The combination chemotherapy regimen FOLFIRINOX (5-fluorouracil, leucovorin, irinotecan, oxaliplatin) improved the survival of PDAC patients to 11.1 months, compared to 6.8 months with gemcitabine. However, this regimen resulted in increased toxicity [28]. Solvent-based taxanes, docetaxel or paclitaxel, either alone or in combination with gemcitabine, have only shown moderate clinical activity [29,30,31,32,33,34,35]. Awasthi et al. demonstrated the superior activity of nab-paclitaxel compared with gemcitabine or docetaxel and showed improved activity of a nab-paclitaxel plus gemcitabine (nabP/Gem) in PDAC preclinical models [36]. Similarly, the superior therapeutic efficacy of nab-paclitaxel over cremophor solvent-based paclitaxel was observed by others in an orthotopic model of PDAC [37].

Based on the promising clinical activity and improved safety of nab-paclitaxel in breast cancer [38], it was evaluated in PDAC clinical trials. In a phase I/II trial, nabP/Gem showed tolerable adverse effects with substantial antitumor activity, with an overall response rate (ORR) of 48%, median progression-free survival (PFS) of 7.9 months, and median overall survival (OS) of 12.2 months, as first-line therapy in metastatic PDAC patients [39]. In a phase II trial of gemcitabine-refractory advanced PDAC patients, nab-paclitaxel was well tolerated and demonstrated preliminary clinical activity as second-line therapy (ORR 58%, PFS 1.7 months, OS 7.3 months) [40]. A phase III landmark trial (MPACT) comparing the nabP/Gem regimen versus gemcitabine monotherapy in metastatic PDAC patients demonstrated that nabP/Gem was superior to gemcitabine in terms of OS (8.5 vs. 6.7 months), PFS (5.5 vs. 3.7 months), and ORR (23 vs. 7%). These results led to the approval of nabP/Gem as a first-line treatment for metastatic PDAC patients [41]. A phase II study with nabP/Gem vs. nab-paclitaxel plus leucovorin/5-FU showed a PFS rate of 54% vs. 56% after 4 months with a tolerable toxicity profile [42]. A phase II study of untreated metastatic PDAC patients with nabP/Gem plus the HSP27-targeting antisense oligonucleotide apatorsen or placebo exhibited no clinical benefit (PFS 2.7 vs. 3.8 months; OS 5.3 vs. 6.9 months) [43]. In a phase Ib/II study of metastatic PDAC patients, nabP/Gem in combination with pembrolizumab, a PD-1 inhibitor, demonstrated that the regimen was well tolerated and there was a slight improvement in efficacy (PFS 9.1 months; OS 15 months) [44].

Considering the excessive hyaluronan (HA) accumulation in the PDAC tumor microenvironment, pegvorhyaluronidase alfa (PEGPH20), which degrades HA, was evaluated in combination with nabP/Gem. In a phase II trial, this regimen compared to nabP/Gem plus placebo demonstrated improved PFS with the largest improvement in patients with HA-high tumors (PFS 9.2 vs. 5.2 months; OS 11.5 vs. 8.5 mo) [45]. However, in the phase III trial, this regimen increased the ORR (47 vs. 36%) but did not improve OS (11.2 vs. 11.5 months) or PFS (7.1 vs. 7.1 months) [46]. In a single-arm phase II study in advanced PDAC patients, nab-paclitaxel plus S1 followed by S-1 maintenance therapy demonstrated encouraging ORR (53.1%), PFS (6.2 months), OS (13.6 months) and manageable toxicity as first-line therapy [47]. The addition of cisplatin and capecitabine to the nabP/Gem backbone in a phase II trial yielded promising clinical efficacy (6-month disease-free survival rate of 74% vs. 46%) with manageable toxicity [48]. Furthermore, a phase I trial of nabP/Gem in combination with enzalutamide, a novel androgen receptor, exhibited an OS of 9.73 months and PFS of 7.53 months, without any unexpected toxicities [49]. A phase II trial of nabP/Gem in combination with hydroxychloroquine (HCQ), an autophagy inhibitor, in untreated advanced PDAC patients showed no improvement in the primary endpoint of OS at 12 months [50]. However, in a later phase II study of PDAC patients, treatment with preoperative HCQ plus nabP/Gem followed by resection resulted in a greater pathologic tumor response, improved serum biomarker response and evidence of autophagy inhibition and immune activity [51]. In phase II trials, the addition of the Notch signaling inhibitor tarextumab [52], or the insulin-like growth factor-1 receptor and HER3 pathway dual inhibitor istiratumab [53], to nabP/Gem in untreated metastatic PDAC patients did not exhibit any significant clinical improvement. Similarly, the addition of vismodegib, a hedgehog (Hh) signaling inhibitor, to nabP/Gem did not improve clinical efficacy in patients with untreated metastatic PDAC (PFS 5.4 months; OS 9.8 months) [54]. A phase II study of untreated metastatic PDAC patients with nabP/Gem plus the heparin mimetic necuparanib or placebo demonstrated no significant benefit (PFS 5.5 vs. 6.9 months; OS 10.7 vs. 9.9 months) [55]. A phase II study of perioperative nabP/Gem for resectable PDAC showed that it was tolerable, but the primary endpoint of an 85% complete resection rate was not met [56]. As an alternative to FOLFIRINOX, nab-paclitaxel was evaluated in combination with FOLFOX in PDAC patients following surgical resection in phase I [57] and phase II [58] trials. This regimen showed manageable toxicity and significant clinical efficacy with a median disease-free survival of 19.7 months and median OS of 53.5 months [58].

Nivolumab, an immune checkpoint inhibitor (ICI) antibody, in combination with nabP/Gem in advanced PDAC, showed a manageable safety profile without any significant clinical benefit (PFS 5.5 months; OS 9.9 months) in a phase I study [59]. Later, a combination of an agonistic CD40 antibody APX005M (sotigalimab) with nabP/Gem and nivolumab demonstrated good tolerability and clinical activity in a phase Ib study, indicating the potential for this regimen to improve chemotherapy-only clinical activity [60]. Since matrix metalloproteinase 9 (MMP9) expression in the PDAC tumor microenvironment has multiple protumorigenic effects [61], and an anti-MMP9 antibody has antitumor activity in combination with nab-paclitaxel-based chemotherapy in preclinical PDAC models [62], the MMP9 antibody andecaliximab (GS-5745) was evaluated in combination with nabP/Gem in patients with advanced PDAC in a phase I study. This regimen demonstrated a favorable safety profile and clinical activity (PFS 7.8 months; ORR 44.4%) [63]. A phase II study of nabP/Gem versus nabP/Gem followed by FOLFIRINOX induction chemotherapy exhibited similar activity (surgical conversion rate 35.9% vs. 43.9%; OS 18.5 vs. 20.7 months) and safety between the two regimens in locally advanced resectable PDAC patients [64]. A phase III study of nabP/Gem plus ibrutinib, a Bruton’s tyrosine kinase inhibitor, did not improve OS or PFE compared to nabP/Gem in advanced PDAC patients [65]. A phase I/II study of two modified regimens of FOLFIRINOX, replacing either oxaliplatin (Nab-FOLFIRI) or irinotecan (Nab-FOLFOX) with nab-paclitaxel, showed promising clinical activity (PFS 6 vs. 5.6 months; OS 10.2 vs. 10.4 months) in patients with metastatic PDAC [66]. A phase II study of nabP/Gem combination with S1 as neoadjuvant chemotherapy in borderline resectable PDAC with arterial contact demonstrated a 43% response rate, and 96% patients received pancreatectomy. The median PFS and OS were 24.2 and 41 months, respectively [67]. In a phase III trial, algenpantucel-L immunotherapy did not improve survival in patients with borderline resectable or locally advanced unresectable PDAC receiving standard neoadjuvant chemotherapy (FOLFIRINOX or nabP/Gem) and chemoradiation [68]. A phase I/II trial evaluated RX-3117, a small molecule antimetabolite, in combination with nab-paclitaxel and demonstrated tolerability, safety and preliminary efficacy (ORR 23.1%, PFS ~5.6 months) in newly diagnosed metastatic PDAC patients [69]. In a phase II trial, sotigalimab and/or nivolumab were evaluated in combination with nabP/Gem in metastatic PDAC patients. The primary endpoint of 1-year OS was met for nivo/chemo (57.7%, *p* = 0.006 compared to a historic 1-year OS of 35%) but was not met for sotiga/chemo (48.1%, *p* = 0.062) or sotiga/nivo/chemo (41.3%, *p* = 0.223) [70]. A phase II trial of nabP/Gem with or without ICIs durvalumab (PD-L1 antibody) and tremelimumab (CTLA-4 antibody) showed no improvement in survival (PFS 5.5 vs. 5.4 months; OS 9.8 vs. 8.8 months) in metastatic PDAC patients [71]. A phase I trial of dual αV-integrin and neuropilin-1 targeting peptide CEND-1 in combination with nabP/Gem for the treatment of untreated metastatic PDAC patients demonstrated an acceptable safety profile, with no dose-limiting toxicities and encouraging activity (median OS 13.2 months) [72]. A phase II trial of neoadjuvant nabP/Gem for borderline resectable PDAC with arterial involvement showed the safety and effectiveness of this regimen, providing a chance for curative resection and improved survival [73]. A phase Ib study of ulixertinib, a novel ERK inhibitor, with nabP/Gem in untreated metastatic PDAC showed potential efficacy (median PFS 5.46 months; OS 12.23 months), a similar frequency of grade ≥ 3 toxicities, but a high rate of all-grade toxicities [74]. Recently, a phase II trial of mFOLFIRINOX versus nabP/Gem in advanced PDAC reported nabP/Gem as a better regimen than mFOLFIRINOX because of its better RR (42.1% vs. 30.9%), CA19-9 response rate (85% vs. 57.1%) and mild gastrointestinal toxicities. Both regimens displayed higher efficacy in the 1-year OS (82.5% and 77.4%) than in the historic data of gemcitabine monotherapy [75]. Phase II and III clinical trials of nab-paclitaxel in PDAC are summarized in Table 1.

Mechanisms of chemoresistance in PDAC involve the heterogeneous stroma, consisting of fibroblasts, myofibroblasts, immune cells, blood vessels, extracellular matrix (ECM), and soluble proteins. The dense desmoplastic stroma acts as a physical barrier for drug delivery, exacerbated by its hypovascular nature [76]. Factors such as pancreatic stellate cells (PaSC), SPARC, hyaluronan, Hedgehog signaling, cytokines, and growth factors contribute to stromal density and hinder drug delivery [77,78,79,80]. PDAC cells produce angiogenic and anti-angiogenic factors, including angiostatin, endostatin, and pigment epithelium-derived factor, which strongly inhibit angiogenesis [81]. Strategies targeting the fibrotic stroma to reverse hypovascularity and enhance drug delivery have been studied. While some stromal-targeted therapies improve drug delivery and patient survival [78,82], trials focused on the Hedgehog signaling pathway showed no clinical benefits [83]. Additionally, hypoxia, a common feature of PDAC, enhances resistance to cytotoxic drugs by slowing the cell cycle and activating hypoxia-inducible factor-1 (HIF-1) to promote cancer cell survival [81]. In the PDAC tumor microenvironment (TME), exosomes play a critical role in chemoresistance, with cancer-associated fibroblasts and cancer stem cells transferring resistance via exosomes [84,85]. Moreover, PDACs can acquire chemoresistance through the uptake of exosomes. At the molecular level, gemcitabine relies on human nucleoside transporters (NTs), particularly hENT1, for effective transport into tumor cells. Deficiency or inhibition of hENT1 leads to gemcitabine resistance [86]. Enzymatic processes, including phosphorylation, deamination, and dephosphorylation, influence gemcitabine’s efficacy, with enzymes like cytidine deaminase (CDA), deoxycytidylate deaminase (dCTD), and 5′-nucleotidases (5′-NTs) affecting its metabolism. These molecular mechanisms impact gemcitabine’s cytotoxicity and overall effectiveness [80,87]. Understanding these mechanisms is crucial for developing strategies to overcome chemoresistance in PDAC.

The chemoresistance mechanisms of nab-paclitaxel in PDAC are similar to those of paclitaxel, and its use in various types of cancer suggests several possible mechanisms. Several major proteins involved in multidrug resistance, including P-glycoprotein (P-gp), also known as multidrug resistance protein 1 (MDR1), MDR-associated protein 1 (MRP1), and breast cancer resistance protein (BCRP), are involved in resistance to all taxanes [88,89]. The impact of paclitaxel on microtubules can lead to alterations in the β-tubulin family within resistant cancer cells. Notably, overexpression of β-tubulin III in PDAC has been linked to increased tumor growth and metastasis [90,91]. Additionally, the gene HE4 (human epididymis protein 4) has been shown to promote PDAC cell growth and reduce sensitivity to paclitaxel, indicating that HE4 expression may be used to predict the sensitivity of PDAC patients to paclitaxel [92]. Recent findings have revealed that sustained induction of c-MYC is associated with nab-paclitaxel resistance in primary PDAC cells [93]. Moreover, exosomes have been shown to influence multidrug resistance proteins involved in paclitaxel resistance [94].

## 4. Nab-Paclitaxel in Esophageal Cancer

There are two main subtypes of esophageal cancer (EC): esophageal adenocarcinoma (EAD) and esophageal squamous cell carcinoma (ESCC) [95]. ESCC, which is more common in Asian countries, accounts for about 90% of the EC worldwide, whereas EAC is the leading type of EC in the USA [96]. In our preclinical studies of esophageal cancer, we observed a higher effectiveness for nab-paclitaxel compared to sb-paclitaxel [97]. As first-line therapy, nab-paclitaxel’s efficacy and safety were compared in a retrospective study where nab-paclitaxel with cisplatin and sb-paclitaxel with cisplatin were given in metastatic ESCC patients [98]. In this study, 32 patients received two cycles of nab-paclitaxel (125 mg/m^2^) plus cisplatin (75 mg/m^2^) (Nab-TP group) intravenously over a 30-min period with adequate hydration but without any premedication in a 21-day period. Similarly, 43 patients received two cycles of sb-paclitaxel (80 mg/m^2^) and the same dose of cisplatin (sb-TP group) for the same 21-day period with premedication of corticosteroids and antihistamines. The incidence of grade ≥3 peripheral neuropathy, arthralgia, and myalgia were significantly lower in the nab-TP group compared to the sb-TP group (all *p* < 0.05), indicating that nab-TP was generally better tolerated. The incidence of hematological toxicities like anemia, leukopenia, neutropenia, febrile neutropenia, and thrombocytopenia did not differ significantly between the two study arms. However, the comparative efficacy and safety profiles of this study were consistent with those in other reports [99,100,101] where nab-paclitaxel was used as the first-line treatment for metastatic ESCC [99,101]. Additionally, nab-TP demonstrated a higher ORR (50% vs. 30%; *p* = 0.082) and disease control rate (DCR) (81% vs. 65%; *p* = 0.124) than sb-TP. Although the median OS was similar between these two groups, nab-TP resulted in a longer median PFS (6.1 months (95% confidence interval: 5.3–6.9)) than sb-TP (5.0 months (95% confidence interval: 4.4–5.6)) (*p* = 0.029).

The therapeutic applicability of nab-paclitaxel, along with its efficacy and safety profiles, have been tested in a variety of clinical studies which are listed in Table 2. Neoadjuvant chemotherapies and chemoradiotherapies have been used as the standard of care for the treatment of advanced EC [102]. The use of nab-paclitaxel in combination with platinum-based therapy, such as cisplatin, has also been explored as neoadjuvant therapy in locally advanced ESSC [103]. In this phase II study using nab-paclitaxel plus cisplatin as neoadjuvant therapy, the ORR was 65.7% and the median OS was 37.8 months, demonstrating the promising role of nab-paclitaxel as an alternative treatment [103]. In this study, no treatment-related death was reported among patients. When comparing treatment-related toxicities, grade ≥ 3 neutropenia was higher (61.9% vs. 11.5%) with a greater risk of postoperative death in a comparable study where sb-paclitaxel was used instead of nab-paclitaxel [104]. The therapeutic efficacy and safety of nab-paclitaxel in combination with ICIs have recently been evaluated as neoadjuvant therapies for advanced EC [105,106,107,108,109]. In these studies, patients received preoperative nab-paclitaxel in combination with ICIs such as camrelizumab [105,106,108,109,110] or tislelizumab [107]. Some treatment-related adverse events (TRAEs) were reported in these studies, but none of them were previously unreported toxicities. Additionally, very few or no patients experienced grade 3 or higher TRAEs. There were no treatment-related perioperative or postoperative deaths reported. A very strong antitumor response was observed with nab-paclitaxel plus camrelizumab, as evidenced by more than 20% to 50% of patients having a complete pathological response. There was also evidence of the complete disappearance of the primary tumors in a significant number of cases, with an enhanced ORR in the nab-paclitaxel plus camrelizumab arms. When comparing with sb-paclitaxel, the complete pathological response was significantly higher in the ICIs plus nab-paclitaxel group compared to the ICIs plus sb-paclitaxel group (36.7% vs. 21.4%, *p* = 0.018) [109].

## 5. Nab-Paclitaxel in Gastric Cancer

Gastric adenocarcinoma (GAC) is the fifth most common cancer and the fourth leading cause of cancer-related death worldwide [96]. For unresectable metastatic or recurrent GAC, combination chemotherapy regimens demonstrate a dismal clinical benefit, leading to a median survival of less than a year [121,122,123,124]. The FLOT (5-FU/leucovorin, oxaliplatin and docetaxel) regimen demonstrated better OS (50 months) compared with the ECF/ECX (epirubicin, cisplatin, fluorouracil or capecitabine) group (35 months) as a perioperative therapy for GAC patients with locally advanced, resectable tumors [125]. Thus, the FLOT regimen is now the standard regimen for a perioperative strategy of resectable GAC patients and is widely utilized for metastatic GAC as well. Meta-analyses indicate that GAC patients’ survival can be improved by second-line therapy after failing first-line chemotherapy [126,127]. Second-line therapy for GAC patients usually involves cytotoxic chemotherapy agents such as taxanes and irinotecan or the two molecular targeted agents trastuzumab and ramucirumab [128]. The median OS of GAC patients receiving second-line therapy ranges from 3.6 to 10.9 months [112,129,130]. In GAC preclinical models, Awasthi et al. recently demonstrated that *nab*-paclitaxel and liposomal irinotecan (*nal*-IRI) have higher anti-tumor efficacy than their solvent-based formulations [131,132,133,134,135]. Due to the low response rates of current standard therapies, and the development of chemoresistance and toxicity [136], several clinical studies evaluated nab-paclitaxel in GAC (Table 2).

A phase II study of nab-paclitaxel (given every 3 weeks) showed promising activity in previously treated unresectable or recurrent GAC (ORR 28 = 7.8%, PFS 2.9 months, OS 9.2 months), with well-tolerated toxicities [111]. A phase I study of nab-paclitaxel in combination with S-1 in unresectable or recurrent GAC patients demonstrated preliminary clinical activity with good tolerability [24]. A phase III trial evaluated nab-paclitaxel versus sb-paclitaxel in previously treated advanced GAC patients. This trial showed that weekly nab-paclitaxel was not inferior to weekly sb-paclitaxel in terms of OS and recommended nab-paclitaxel as a potential second-line GAC therapy based on the other advantages associated with this regimen [112]. A phase II study of nab-paclitaxel plus ramucirumab in previously treated advanced GAC patients demonstrated promising activity with manageable toxicities. In this trial, the ORR was 54.8% and PFS was 7.6 months [112]. A phase II study of tri-weekly low-dose nab-paclitaxel (180 mg/m^2^) showed clinical efficacy (ORR 5.9%, median PFS 2.4 months and OS 9.2 months) in advanced GAC patients with good tolerability [113]. A phase III trial showed that peritoneal metastasis might be a predictive factor for nab-paclitaxel second-line treatment of advanced GAC patients [114]. A triple chemotherapy combination of nab-paclitaxel, oxaliplatin and 5-FU as a perioperative regimen in resectable GAC patients demonstrated promising activity with 95.5% of patients having R0 tumor resection, but there was a significant rate of surgical complications [137]. A phase II trial of nab-paclitaxel as a second-line therapy for fluoropyrimidine-refractory advanced GAC patients demonstrated a manageable safety profile and significant clinical activity with a median PFS of 3.5 months, OS of 9 months, ORR of 16% and DCR of 72% [115]. In Japanese patients, a reduced dose of nab-paclitaxel (tri-weekly 220 mg/m^2^) was not recommended as a second-line therapy in advanced or recurrent GAC based on its dismal response rate [116].

## 6. Nab-Paclitaxel in Cholangiocarcinoma

Cholangiocarcinoma (CCA) is a highly aggressive biliary tract cancer (BTC). Depending on their anatomical site of origin, CCAs are divided into three subtypes: intrahepatic (iCCA, 10–20%), perihilar (pCCA, 50–60%), and distal (dCCA, 20–30%) [138]. CCA has a very poor prognosis with a 5-year survival rate of 5–15% [139]. Complete surgical resection is the only chance for long-term survival, but unfortunately, most CCAs are diagnosed with metastatic or locally advanced unresectable disease [140]. For unresectable and recurrent CCA, combination chemotherapy with gemcitabine and cisplatin (GemCis) remains the standard treatment despite moderate clinical activity, as demonstrated by the median survival of only 14 months [141]. Nab-paclitaxel is currently under clinical investigation for CCA based on its superior antitumor efficacy and safety profile in other solid tumors (Table 2).

Nab-paclitaxel plus gemcitabine was evaluated as a first-line treatment of advanced and metastatic CCA in a phase II trial. This regimen was well tolerated but did not meet its primary efficacy endpoint [117]. Considering historical data on PFS of 8.0 months and an OS of 11.7 months with the GemCis regimen [142], a phase II trial of gemcitabine, cisplatin and nab-paclitaxel combination demonstrated significant clinical activity by prolonging PFS (11.8 months) and OS (19.2 months) [118]. A phase III trial of this regimen in treating patients with newly diagnosed advanced CCA is currently ongoing (NCT03768414). A single-arm phase I study of nab-paclitaxel plus capecitabine as a second-line therapy in GemCis refractory advanced CCA patients demonstrated adequate safety and promising early efficacy with median PFS and OS of 5.7 and 12.1 months, respectively [143].

## 7. Nab-Paclitaxel in Colorectal Cancer

Colorectal cancer (CRC) is the third biggest cause of cancer-associated death and the fourth most frequently diagnosed cancer in the world [144]. Even though taxanes are widely used for the treatment of upper GI tract (esophageal and gastric) cancers, their use in the treatment of lower GI tract (colorectal) cancers is very limited due to poor response and/or development of resistance, although the mechanism of this resistance is not clearly known [145]. Resistance to paclitaxel in CRC can be related to P-gp, as it is overexpressed in CRC [146,147], and a higher dose of paclitaxel has been associated with overcoming the paclitaxel resistance [148]. Thus, there is a therapeutic potential of using nab-paclitaxel to overcome paclitaxel resistance in CRC, as an enhanced accumulation of nab-paclitaxel can be achieved inside CRC xenografts compared to sb-paclitaxel [9]. In addition, the enhanced in vivo antitumor effect of nab-paclitaxel, overcoming sb-paclitaxel resistance, is attributed to its antiangiogenic mechanism [149]. However, a multicenter phase II clinical trial of nab-paclitaxel monotherapy in previously treated metastatic CRC (mCRC) patients did not show an encouraging anticancer effect [119]. Interestingly, in another phase II clinical trial, nab-paclitaxel monotherapy exhibited improvement in median PFS in small bowel adenocarcinoma compared to CRC having a high frequency of genome methylation at CpG islands (CIMP-high CRC) (3.2 months vs. 2.1 months, *p =* 0.03) [120]. Taxane resistance has been linked to CIMP-high CRC patients [150], and nab-paclitaxel monotherapy did not improve clinical outcomes in these patients, suggesting that CIMP-high should not be used as a predictive biomarker for nab-paclitaxel monotherapy. On the other hand, nab-paclitaxel at a dose of 200 mg/m^2^ for 21 days proved to be safe in intestinal cancer patients. Adverse effects were reported in less than 10% of the patients, with no report of grade 5 adverse effects. Thus, nab-paclitaxel monotherapy may not be an effective therapeutic option for treating mCRC, and nab-paclitaxel in combination with another anticancer drug may be more effective. Results from ongoing or recently completed clinical trials (NCT03563157, NCT01730586, NCT02574663, NCT02857270) where nab-paclitaxel has been used in combination with other chemotherapies or targeted therapies for the treatment of CRC may provide answers regarding the role of nab-paclitaxel in CRC combination therapies. The comparative role of nab-paclitaxel as mono and combination therapies in CRC has been summarized in Table 2.

## 8. Future Prospective and Conclusions

Nab-paclitaxel has shown superior activity to paclitaxel in many types of cancers, including GI cancers. However, clinical response to nab-paclitaxel can be hampered by resistance development, and the associated mechanism is not well studied. SPARC is a secreted protein that is overexpressed in multiple cancers, including GI cancers, and its overexpression may be associated with a positive response to nab-paclitaxel [151]. As SPARC is an albumin-binding protein, it is thought to facilitate the intratumoral accumulation of nab-paclitaxel [9]. However, the role of SPARC as a predictive biomarker in GI cancers is not very clear [152]. Therefore, further studies are needed to determine the potential role of novel predictive biomarkers in assessing the nab-paclitaxel response in GI cancers.

The nanoparticle albumin-bound treatment strategy is an encouraging method for delivering other water-insoluble anticancer drugs. Drugs like docetaxel and the mTOR (mammalian target of rapamycin) inhibitor rapamycin have been formulated as nanoparticles in solvent-free drug formulations. A phase I clinical trial (NCT04931823) is currently recruiting patients to study the safety of nab-docetaxel (CPO-100) in advanced solid tumors. Similarly, nab-sirolimus (nab-rapamycin) is currently being investigated in a phase I clinical trial (NCT05661461) involving various solid tumors. These novel nanoparticle albumin-bound drugs will hopefully display higher tumor penetration and anti-tumor activity in various cancers, including GI cancers.

In conclusion, nab-paclitaxel has demonstrated improved clinical activity and safety compared to sb-paclitaxel in most GI cancers. While nab-paclitaxel is currently approved for metastatic PDAC among all GI cancers, its efficacy and safety in other GI cancers are being evaluated in clinical trials. Based on encouraging results from these clinical trials, the utilization of nab-paclitaxel in different GI cancers is expected to increase in the future. Thus, nab-paclitaxel has the potential to become part of clinical regimens to improve the therapy of most GI cancer patients.

## Figures and Tables

**Table 1:** Phase II and III clinical trials of nab-paclitaxel in PDAC.

Authors,	Patients	Therapeutic	ORR	Median	Median	Common
Year		Regimen	(%)	PFS (Mo)	OS (Mo)	Adverse Events %
						Hematologic (Grade ≥ 3)
Von Hoff et al., 2011 [39]	Untreated advanced	NG	48	7.9	12.2	neutropenia 67 leukopenia 44 Thrombocytopenia 23
Hosein et al., 2013 [40]	Gemcitabine refractory	nab-pac	58	1.7	7.3	neutropenia 32 anemia 11
Von Hoff et al., 2013 [41]	Untreated metastatic	NG vs. gemcitabine	23 vs. 7	5.5 vs. 3.7	8.5 vs. 6.7	neutropenia 38 vs. 27 leukopenia 31 vs. 16 thrombocytopenia 13 vs. 9 anemia 13 vs. 12
Bachet et al., 2017 [42].	untreated metastatic	NG vs nab-pac+ leucovorin/5-FU		at 4 m PFS rate 54% vs. 56%		neutropenia 32 vs. 23 thrombocytopenia 18 (NG anemia 13 (NG)
Ko et al., 2017 [43].	Untreated metastatic	NG+apatorsen vs NG	18 for both	2.7 vs. 3.8	5.3 vs. 6.9	leukopenia 0 (both) thrombocytopenia 0 (both)
Weiss et al., 2018 [44].	untreated metastatic	NG+ pembrolizumab		9.1	15	neutropenia 47 thrombocytopenia 20
Hingorani et al., 2018 [45]	untreated metastatic	NG+PEGPH20 vs NG	In high hyaluronan patients			neutropenia 29 vs. 18
			45 vs. 31	9.2 vs. 5.2	11.5 vs. 8.5	thrombocytopenia 16 vs. 9
Cutsem et al., 2020 [46].	untreated Metastatic High hyaluronan	NG+PEGPH20 vs. NG	47 vs. 36	7.1 vs. 7.1	11.2 vs. 11.5	thrombocytopenia 21 vs. 16
Zhang et al., 2018 [47].	untreated advanced	nab-pac+ S1 then S1 maintenance	53.1	6.2	13.6	neutropenia 27.6
Reni et al., 2018 [48].	untreated metastatic	NG+cisplatin+ capecitabine vs. NG		at 6 months DFR 74 vs. 46%		neutropenia 41 vs. 39 anemia 21 vs. 22
Karasic et al., 2019 [50].	untreated advanced	NG+HCQ vs NG	38.2 vs. 21	5.7 vs. 6.4	11.1 vs. 12.1	neutropenia 42.6 vs. 22.6 anemia 3.7 vs. 17
Zeh et al., 2020 [51].	Untreated resectable	preoperative NG+ HCQ vs. NG		recurrence-free survival 16.6 vs. 13.5	36 vs. 32	all grade ≥ 3 adverse events 62.5 vs. 60.5
Hu et al., 2019 [52].	Untreated metastatic	NG+ Tarextumab vs NG	20.2 vs. 31.8	3.7 vs. 5.5	6.4 vs. 7.9	neutropenia 9 vs. 18 thrombocytopenia 49 vs. 25 anemia 29 vs. 26
Kundranda et al, 2020 [53].	untreated metastatic	NG+ Istiratumab vs NG	39.5 vs. 51.2	high IGF-1 3.6 vs. 7.3 high IGF-1/HRG+ 4.1 vs. 7.3	8.9 vs. 11.7	neutropenia 30 vs. 34 anemia 18.6 vs. 18.2 thrombocytopenia 16 vs. 7
De Jesus-Acosta et al, 2020 [54].	untreated metastatic	NG+ vismodegib	40	5.4	9.8	No data about neutropenia, anemia or thrombocytopenia
O’Reilly et al., 2020 [55].	Untreated metastatic	NG+ Necuparanib vs NG		5.5 vs. 6.9	10.7 vs. 9.99	neutropenia 33 vs. 34 thrombocytopenia 27 vs. 5 anemia 22 vs. 11
Barbour et al., 2020 [56].	resectable	Perioperative NG		12.3	23.5	neutropenia 40 anemia 10 thrombocytopenia 5
Raufi et al., 2020 [58].	resected	adjuvant FOLFOX plus nab-pac		19.7	53.5	neutropenia 26
Kunzmann et al, 2021 [64].	locally advanced resectable	NG vs. NG followed by FOLFIRINOX	surgical conversion rate 35.9% vs. 43.9%		18.5 vs. 20.7	neutropenia 28 vs. 24
Tempero et al, 2021 [65].	untreated metastatic	NG+ibrutinib vs. NG	29 vs. 42	5.3 vs. 6	9.7 vs. 10.8	neutropenia 24 vs. 35 anemia 16 vs. 17
Giommoni et al., 2021 [66].	untreated metastatic	nab-pac+FOLFIRI vs nab-pac+FOLFOX	31 for both	6 vs. 5.6	10.2 vs. 10.4	neutropenia 19 vs. 29 febrile neutropenia 12 vs. 1 thrombocytopenia 2.4 vs. 0 anemia 7 vs. 10
Kondo et al., 2021 [67].	borderlineresectable arterial contact	Neoadjuvant NG+S-1	43	24.2	41	neutropenia 25 leukopenia 19 anemia 2
Hewitt et al, 2022 [68].	Borderline resectable or locally advancedunresectable	soc [(FOLFIRINOX or NG) plus chemoradition] plus algenpantucel vs soc + placebo		12.4 vs. 13.4	14.3 vs. 14.9	all grade ≥ 3 adverse events 81 vs. 75
Babiker et al., 2022 [69].	untreated metastatic	nab-pac plus RX-3117	23.1	5.6		neutropenia 20 anemia 22
Padron et al., 2022 [70].	untreated metastatic	NG+nivolumabNG+sotigalimab NG+sotiga+nivo	50 vs. 33 vs 31	6.4 vs. 7.3 vs. 6.7	16.7 vs. 11.4 vs. 10.1	neutropenia 33, 54, 57 thrombocytopenia 11, 16, 60 anemia 33, 24, 51
Renouf et al., 2022 [71].	untreated metastatic	NG+ durvalumab +tremelimumabvs NG	30.3 vs. 23	5.5 vs. 5.4	9.8 vs. 8.8	neutropenia 49 vs. 44 thrombocytopenia 11 vs. 16 anemia 22 vs. 32 lymphopenia 38 vs. 20
Ikenaga et al., 2023 [73].	borderline resectable with arterial involvement	Neoadjuvant NG			24.9	neutropenia 68 leukopenia 39 thrombocytopenia 7 anemia 4
Ozaka et al., 2023 [75].	untreated locally Advanced	mFOLFIRINOXvs NG	30.9 vs. 42.1	11.2 vs. 9.4	23 vs. 21.3	neutropenia 60 vs. 79 leukopenia 23 vs. 44 anemia 11 vs. 19

PDAC, pancreatic ductal adenocarcinoma; NG, nab-paclitaxel plus gemcitabine; ORR, overall response rate; PFS, progression-free survival; OS, overall survival; mo, months; vs, versus; nab-pac, nab-paclitaxel, 5-FU, 5-fluorouracil; PEGPH20, pegvorhyaluronidase alfa; HCQ, hydroxychloroquine; FOLFIRINOX, 5-fluorouracil/leucovorin plus irinotecan plus oxaliplatin; FOLFIRI, folinic acid plus 5-fluorouracil plus irinotecan; FOLFOX, folinic acid plus 5-fluorouracil plus oxaliplatin; soc, standard of care.

**Table 2:** Phase II and III clinical trials of nab-paclitaxel in esophageal, gastric, bile duct and colorectal cancers.

Authors,	Patients	Therapeutic	ORR	Median	Median	Common
Year		Regimen	(%)	PFS (Mo)	OS (Mo)	Adverse Events %
						Hematologic (Grade ≥ 3)
Yun Fan et al., 2016 [103].	neoadjuvantlocally advancedESCC	Nab-paclitaxel + cisplatin	ORR 65.7%	34.7	37.8	neutropenia 11.5 anemia 8.6 thrombocytopenia 5
Guozhen Yang et al, 2021 [105].	neoadjuvant locally advancedESCC	Nab-paclitaxel + camrelizumab + S1	33.33% (cPR)	no surgicaldelay	no preoperativedeath	neutropenia 0 anemia 0 thrombocytopenia 0
Jun Liuet al, 2022 [106].	neoadjuvantlocally advancedESCC	Nab-paclitaxel+ carboplatin+ camrelizumab	39.2% (cPR)	no surgicaldelay	no preoperativedeath	neutropenia 50 anemia 6.7 thrombocytopenia 6.7
Yafan Yang et al., 2023 [109].	neoadjuvant locally resectable ESCCICIs+ paclitaxel + cisplatin	ICIs+ Nab-paclitaxel+ cisplatin vs.	36.7% (cPR) vs. 21.4% (cPR)	PFS not yet reached	OS not yet reached	not yet reached
YasutsunaSasaki et al., 2014 [111].	previously treated resectable or recurrent GC	Nab-paclitaxel	ORR 27.8%	2.9	9.2	neutropenia 49.1 anemia 7.3 thrombocytopenia 0
Hideaki Bonda et al., 2018 [112].	previouslytreated advanced GC	Nab-paclitaxel+ ramucirumab	ORR 54.8%	7.6	not yet reached	neutropenia 76.7 anemia 11.6
Sho Sato et al., 2018 [113].	previously treatedunresectable or recurrent GC	Nab-paclitaxel	ORR 5.9%	2.4	9.2	neutropenia 5.9 anemia 8.8
Atsuo Takashima et al., 2019 [114].	pretreated advancedPM GC	Nab-paclitaxel vs. sb-paclitaxel	-------	4.0 vs. 2.6	7.6 vs. 4.9	--------
DaisukeKobayashi et al., 2020 [115].	previously treated advanced GC	Nab-paclitaxel	ORR 16%	3.5	9.0	neutropenia 49 anemia 2 thrombocytopenia 0
ShegeyukiTamura et al, 2020 [116].	previouslytreatedunresectable or recurrent GC	Nab-paclitaxel (low dose)	ORR 3.1%	2.2	6.3	neutropenia 37.5 anemia 12.5 thrombocytopenia 3.1
VaibhavSahaiet al, 2018 [117].	First line therapyadvanced or metastatic CCA	Nab-paclitaxel+ gemcitabine	ORR 30%	7.7	12.4	neutropenia 43 anemia 15 thrombocytopenia 16
Rachna Shroff et al., 2019 [118].	First line therapy advanced BTCs	Nab-paclitaxel + gemcitabine + cisplatin	PRR 45%	11.8	19.2	neutropenia 33 anemia 16 thrombocytopenia 13
Michel Ducreux et al., 2017 [119].	pretreatedmCRC	Nab-paclitaxel	ORR 0%	8.1 weeks	--------	neutropenia 22 anemia 5
Overman et al., 2018 [120].	pretreated refractory SBAvsCIMP high CRC	Nab-paclitaxel	ORR 20% vs. ORR 0%	3.2 vs. 2.1	not yet reached	neutropenia 9 anemia 0 thrombocytopenia 6

Abbreviations: ORR (overall response rate), cPR (complete pathological response), PFS (progressive free survival) OS (overall survival), ESCC (esophageal squamous cell carcinoma), GC (gastric cancer), PM GC (peritoneal metastasis gastric cancer), CCA (cholangiocarcinoma), BTCs (Biliary tract cancers), PRR (partial response rate), mCRC (metastatic colorectal cancer), SBA (small bowel adenocarcinoma), CIMP (CpG island methylator phenotype), CRC (colorectal cancer).

## Data Availability

The data that support the findings of this study are available from the corresponding author upon reasonable request.
